# Vertical pons hyperintensity and hot cross bun sign in cerebellar-type multiple system atrophy and spinocerebellar ataxia type 3

**DOI:** 10.1186/s12883-020-01738-9

**Published:** 2020-04-27

**Authors:** Atsuhiko Sugiyama, Hajime Yokota, Yoshitaka Yamanaka, Hiroki Mukai, Tatsuya Yamamoto, Shigeki Hirano, Kyosuke Koide, Shoichi Ito, Satoshi Kuwabara

**Affiliations:** 1 Department of Neurology, Graduate School of Medicine, Chiba University, Chiba, Japan; 2 Department of Diagnostic Radiology and Radiation Oncology, Graduate School of Medicine, Chiba University, Chiba, Japan; 3 Department of Rehabilitation, Division of Occupational Therapy, Chiba Prefectural University of Health Sciences, Chiba, Japan; 4 Department of Medical Education, Graduate School of Medicine, Chiba University, Chiba, Japan

**Keywords:** Multiple system atrophy, Magnetic resonance imaging, Hot cross bun sign, Spinocerebellar ataxia type 3, Orthostatic hypotension

## Abstract

**Background:**

The “hot cross bun” (HCB) sign, a cruciform hyperintensity in the pons on magnetic resonance imaging (MRI), has gradually been identified as a typical finding in multiple system atrophy, cerebellar-type (MSA-C). Few reports have evaluated the sensitivity of an HCB, including a cruciform hyperintensity and vertical line in the pons, which precedes a cruciform hyperintensity, in the early stages of MSA-C. Moreover, the difference in frequency and timing of appearance of an HCB between MSA-C and spinocerebellar ataxia type 3 (SCA3) has not been fully investigated.

**Methods:**

This study investigated the time at which an HCB and orthostatic hypotension (OH) appeared in 41 patients with MSA-C, based on brain MRI and head-up tilt test. The MRI findings were compared with those of 26 patients with SCA3. The pontine signal findings on T2-weighted MRI were graded as 0 (no change), 1 (a vertical T2 high-intensity line), or 2 (a cruciform T2 high-intensity line), with grades 1 or 2 considered as an HCB. OH 30/15 was defined as a decrease in systolic blood pressure of > 30 mmHg or diastolic blood pressure of > 15 mmHg.

**Results:**

Among the 24 patients with MSA-C within 2 years from the onset of motor symptoms, an HCB was detected in 91.7%, whereas OH 30/15 was present in 60.0%. Among the 36 patients with MSA-C within 3 years from the onset of motor symptoms, a grade 2 HCB was detected in 66.7% of those with MSA-C but in none of those with SCA-3.

**Conclusions:**

HCB is a highly sensitive finding for MSA-C, even in the early stages of the disease. A grade 2 HCB in the early stage is an extremely specific finding for differentiating MSA-C from SCA-3.

## Background

Multiple system atrophy (MSA) is a progressive, adult-onset neurodegenerative disorder characterized by various combinations of autonomic failure, parkinsonism, cerebellar ataxia, and pyramidal signs. The current consensus criteria, which were revised in 2008, have been widely accepted as the diagnostic guideline for MSA [1]. In the second consensus criteria, autonomic failure, including orthostatic hypotension (OH) and/or urogenital symptoms, is an essential clinical parameter [1]. However, the second consensus criteria are not sensitive for the diagnosis of early-stage MSA. A validation study on the second consensus criteria showed that the sensitivity for probable MSA at the first visit was only 18% and that for possible MSA based on the first consensus was 41% [2]. Therefore, it is important to overcome the poor sensitivity of the second consensus criteria in MSA diagnosis during the early stages of the disease.

In the second consensus criteria, magnetic resonance imaging (MRI) does not play a major role in diagnosis. Atrophy of the putamen, middle cerebellar peduncle, pons, or cerebellum on MRI was only included as an additional feature of possible MSA [1]. However, other conventional MRI features have been widely used to diagnose MSA or to rule out other possible diagnoses [3]. The “hot cross bun” (HCB) sign, that is, a cruciform hyperintensity in the pons on T2-weighted imaging (T2WI), was named for the Easter pastry it resembles and was reported to be a typical feature of MSA [4, 5]. Initially, only a vertical line is seen, but with disease progression, it becomes cruciform with the addition of a horizontal line [6, 7]. In the early stages of the disease, a cruciform hyperintensity in the pons is not very sensitive, and a Japanese study with a large cohort revealed that a cruciform hyperintensity was detected in 64.0% of patients with MSA-C who underwent brain MRI within 2 years after the onset of motor impairment [8]. Although focusing on a vertical line preceding a cruciform hyperintensity in the pons may increase sensitivity, few reports have examined the sensitivity of a vertical hyperintensity in the pons in the early disease stage.

One reason why the HCB is not included in the current consensus criteria [1] may be that the sign is not specific to MSA. It can also be observed in various forms of spinocerebellar ataxia (SCA), including SCA1, 2, 3, 7, 8, and 34 [9, 10]. SCA3 is a common autosomal dominant cerebellar ataxia that occurs in most populations [11]. Moreover, in Japan, SCA3 is the most common SCA in which the HCB can be observed [12]. Hence, identifying differences between MSA and SCA3 is important in progressive ataxia cases in which an HCB is seen on brain MRI, especially in Japan. As disease progression rates of MSA and SCA-3 differ [8, 13], the timing of HCB appearance is expected to differ between MSA and SCA-3. However, the difference between MSA and SCA3 in terms of the frequency and timing of appearance of the HCB has not been fully investigated.

We hypothesized that a vertical hyperintensity in the pons is a highly sensitive MRI finding in the early disease course of MSA-C and that the appearance of an HCB early in the disease course is a specific MRI finding for differentiating MSA-C from SCA3. This study compared the frequency and timing of HCB appearance in the disease course between patients with MSA (either parkinsonian or cerebellar) and SCA3.

## Methods

### Subjects

This retrospective study was approved by the institutional review board of our institution, and the need for informed consent was waived. Inclusion criteria for patients with MSA were as follows: admission between April 2010 and December 2017, having undergone 1.5 T MRI, and clinically confirmed probable MSA based on the diagnostic categories in the second consensus statement [1]. Diagnosis of probable MSA was confirmed by a movement disorder specialist at our center.

In total, 81 patients with MSA were identified, of whom 1 was excluded because of a previous history of putaminal hemorrhage, leaving 80 included in the study. One of the 80 patients underwent postmortem examination of the brain, and the diagnosis was pathologically confirmed. The inclusion criteria for patients with SCA3 were a genetically confirmed SCA3 diagnosis, an examination at our institution between December 2003 and June 2018, and having undergone a 1.5 Tesla MRI test. A total of 26 patients with SCA3 were identified, and none were excluded because of a history of any other central nervous system disorder. Conversely, the inclusion criteria for control subjects were consecutive patients who were referred to our hospital with complaints of headache or dizziness and had no neurologic abnormalities and who had undergone a 1.5 Tesla MRI test between October 2007 and March 2018. In total, 22 control subjects were included in the study.

At the time of diagnosis of probable MSA, it was classified according to whether the clinical syndrome was dominated by parkinsonism (MSA-P) or cerebellar ataxia (MSA-C). The medical records of all patients were reviewed for age at onset, age at MRI, and disease duration from onset to MRI. Disease onset was defined as the initial presentation of any motor problem. The results of head-up tilt tests were reviewed in patients with MSA.

### Image interpretation

All MRI examinations had been performed as part of routine clinical care, and T2WI data were available for all patients. Among the 80 patients with MSA, 125 MRI scans were performed. Among the 26 patients with SCA3, 50 MRI results were available for evaluation, whereas among the 22 control subjects, 22 MRIs were available for evaluation. Two board-certified neuroradiologists (H.Y. and H.M., with 13 and 11 years of experience, respectively) who were blinded to the clinical data independently evaluated the MRIs for each subject. When interpretations differed, the final result was determined by consensus of the two neuroradiologists and a board-certified neurologist (A.S., with 11 years of experience).

**Fig. 1:**
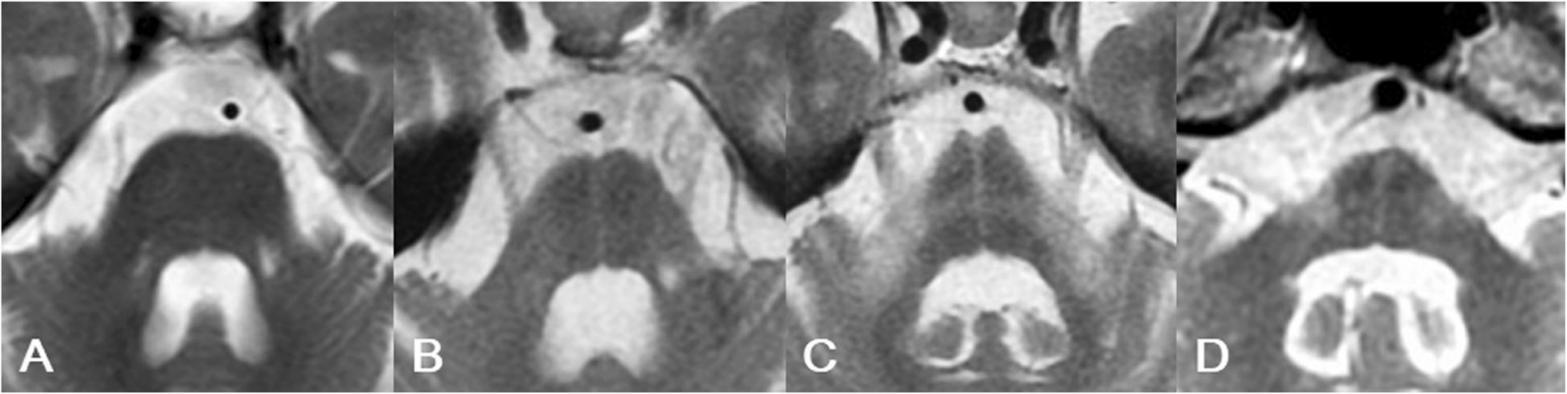
Three grades of the hot cross bun (HCB) sign on magnetic resonance imaging. **a** Grade 0 (negative) HCB sign in a 74-year-old woman with cerebellar-type multiple system atrophy (MSA-C) with a disease duration of 0.3 years. There is no signal change in the ventral pons. **b** Grade 1 HCB sign in a 74-year-old woman with MSA-C with a disease duration of 2.3 years. A vertical T2 high-intensity line is clearly seen in the ventral pons. **c** Grade 2 HCB sign in a 61-year-old man with MSA-C with a disease duration of 2.3 years. Both horizontal and vertical lines are clearly seen in the ventral pons. **d** Grade 2 HCB sign in a 75-year-old woman with SCA3 with a disease duration of 8.2 years. Both horizontal and vertical lines are clearly seen in the ventral pons

We graded the pontine signal findings as 0, with no changes seen; 1, when a vertical T2 high-intensity line began to appear or a clear vertical line was present; and 2, when a horizontal line began to appear along with a vertical line or clear horizontal and vertical lines were seen in the ventral pons (Fig. 1). In this study, both grade 1 and grade 2 were defined as an HCB.

### Assessment for orthostatic hypotension

The head-up tilt test was performed for patients with MSA in a quiet room at an ambient temperature of 22 °C to 26 °C. Each subject lay supine on the table for at least 15 min before the test. Blood pressure and heart rate were measured using a sphygmomanometer at 1-min intervals during the test. After 5 min of baseline measurements, the subject was passively tilted on the electrically driven tilt table to 70° for 10 min. Some patients had more than one tilt table test, so that 120 results for the 80 patients with MSA were available. Based on the diagnostic criteria for probable MSA, “OH 30/15” was defined as a decrease in systolic blood pressure of at least 30 mmHg or a decrease in diastolic blood pressure of at least 15 mmHg within 3 min of tilting [1] and “OH 20/10” was defined as a decrease in systolic blood pressure of at least 20 mmHg or a decrease in diastolic blood pressure of at least 10 mmHg within 3 min of tilting [14].

### Statistical analysis

SPSS software, ver. 25.0 (SPSS Japan, Tokyo, Japan), was used to perform all statistical analyses. The demographic variables of patients with MSA and SCA3 and control subjects were compared using the Kruskal–Wallis one-way analysis of variance with post hoc Mann–Whitney *U* test adjusted for multiple comparisons for age at MRI and frequency of MRI and the χ^2^ test for the gender. However, the demographic variables of patients with MSA or SCA3 were compared using a *t*-test and the Mann–Whitney *U* test for continuous variables. The χ^2^ and Fisher’s exact probability tests were used to compare the prevalence of the HCB sign between patients with MSA-C, SCA3, and controls. The *t*-test and the Mann–Whitney *U* test were used to compare disease duration at the first report of an HCB in patients with MSA-C or SCA3.

## Results

Among the 80 patients with MSA, 41 were classified as having MSA-C and 39 with MSA-P. The demographics of the subject groups included in the study are shown in Table 1. There were no statistically significant differences noted in the sex distribution of the subjects. On comparing patients with MSA and SCA3 and control subjects, it was found that patients with MSA were older than the patients with SCA3 when MRI was performed (*p* = 0.003) and were older than patients with SCA3 at disease onset (*p* < 0.001). Moreover, patients with SCA3 had longer disease duration at the time of MRI than those with MSA (*p* < 0.001). The control subjects underwent MRI tests less frequently than patients with MSA and SCA3 (*p* < 0.001 and *p* = 0.001). On comparing patients with MSA-C and SCA3 and control subjects, it was found that patients with MSA-C were older than the patients with SCA3 at disease onset (*p* < 0.001). Also, patients with SCA3 had longer disease duration at the time of MRI than those with MSA-C (p < 0.001). The control subjects underwent MRI tests less frequently than patients with MSA-C (*p* = 0.001).

**Table 1:** Demographics of the patients with MSA, SCA3, and the controls

	Sex Distribution^a^ (Male/Female)	Age at MRI^b^ (years, median, range)	Age at Onset^c^ (years, mean±SD)	Disease Duration^d^ (years, median, range)	Frequency of MRI^b^ (times, median, range)
MSA (n = 80)	40/40	67.0 (46-82)	63.2 ± 8.7	2.2 (0.2-8.3)	1 (1-5)
MSA-C (n = 41)	21/20	63.5 (46-82)	61.3 ± 9.9	2.1 (0.2-8.3)	1 (1-5)
SCA3 (n = 26)	10/16	58.5 (24-82)	46.0 ± 17.2	6.7 (0.8-24.0)	1 (1-6)
Controls (n = 22)	11/11	64.0 (42-82)	NA	NA	1 (1-1)
*P* value for MSA vs. SCA3	NA	NA	**<0.001**	**<0.001**	NA
*P* value for MSA-C vs. SCA3	NA	NA	**<0.001**	**<0.001**	NA
*P* value for group comparison (MSA, SCA3, Controls)	0.575	**0.01**	NA	NA	**0.002**
*P* value for group comparison (MSA-C, SCA3, Controls)	0.568	0.107	NA	NA	**0.003**
*P* value for post-hoc group comparisons					
MSA vs. SCA3	NA	**0.003**	NA	NA	0.597
MSA vs. Controls	NA	0.235	NA	NA	**<0.001**
SCA3 vs. Controls	NA	0.191	NA	NA	**0.001**
MSA-C vs. SCA3	NA	NA	NA	NA	0.713
MSA-C vs. Controls	NA	NA	NA	NA	**0.001**

^a^Chi-squared test (post-hoc chi-squared tests adjusted for multiple comparison: P < 0.05/3 = 0.0167)

^b^Nonparametric test (Kruskal-Wallis 1-way ANOVA with post-hoc Mann-Whitney *U* test adjusted for multiple comparison: P < 0.05/3 = 0.0167)

^c^Student's t-test

^d^Mann-Whitney *U* test

### Hot cross bun sign in MSA and SCA3

The κ value of the interrater variability between the two examiners who evaluated the HCB was 0.882. The frequencies of HCB signs observed in MSA, SCA3, and controls are listed in Table 2. The frequencies of HCB signs among MSA-C, SCA3, and controls are compared in Table 3. Grade 1 or 2 HCB sign was found in 38 of 41 MSA-C patients (92.7%), 21 of 26 SCA3 patients (80.8%), and none of the controls. Grade 1 or 2 HCB was more frequently observed in MSA-C and SCA3 than in controls (*p* < 0.001). There was no statistical difference in the frequency of grade 1 or 2 HCB between MSA-C and SCA3. A grade 2 HCB sign was found in 30 of 41 MSA-C patients (73.2%), 8 of 26 SCA3 patients (30.8%), and none of the controls. A grade 2 HCB sign was more frequently observed in MSA-C and SCA3 than in controls (*p* < 0.001 and *p* = 0.004), and it was more frequently observed in MSA-C than in SCA3 (*p* = 0.001). Among the patients who underwent MRI within 2 years after disease onset, there was no statistically significant difference in the frequency of a grade 2 HCB sign between MSA-C and SCA3, as was in the frequency of grade 1 or 2 HCB sign between MSA-C and SCA3. Among the patients who underwent MRI within 3 years after disease onset, a grade 2 HCB was more frequently observed in MSA-C than in SCA3 (*p* = 0.001), as was grades 1 or 2 HCB (*p* = 0.014).

**Table 2:** Hot cross bun (HCB) sign on MRI in MSA and SCA3

	Grade 1 or 2 HCB sign (vertical or cruciform hyper-intensity)	Grade 2 HCB sign (cruciform hyper-intensity)
All		
MSA (n = 80)	51/80 (63.8%)	39/80 (48.8%)
MSA-C (n = 41)	38/41 (92.7%)	30/41 (73.2%)
MSA-P (n = 39)	13/39 (33.3%)	9/39 (23.1%)
SCA3 (n = 24)	21/26 (80.8%)	8/26 (30.8%)
Controls (n = 22)	0/22 (0%)	0/22 (0%)
Within two years after onset		
MSA (n = 43)	25/43 (58.1%)	14/43 (32.6%)
MSA-C (n = 24)	22/24 (91.7%)	13/24 (54.2%)
MSA-P (n = 19)	3/19 (15.8%)	1/19 (5.3%)
SCA3 (n = 4)	2/4 (50.0%)	0/4 (0%)
Within three years after onset		
MSA (n = 60)	37/60 (61.7%)	26/60 (43.3%)
MSA-C (n = 36)	33/36 (91.7%)	24/36 (66.7%)
MSA-P (n = 24)	4/24 (16.7%)	2/24 (8.3%)
SCA3 (n = 8)	4/8 (50.0%)	0/8 (0%)

**Table 3:** Hot cross bun (HCB) sign on MRI in MSA-C, SCA3, snd controls

	Grade 1 or 2 HCB sign (vertical or cruciform hyper-intensity)	Grade 2 HCB sign (cruciform hyper-intensity)
MSA-C	38/41 (92.7%)	30/41 (73.2%)
SCA3	21/26 (80.8%)	8/26 (30.8%)
Controls	0/22 (0%)	0/22 (0%)
*P* value for group comparison	**< 0.001**^**a**^	**< 0.001**^**a**^
*p* value for post-hoc group comparisons		
MSA-C vs. SCA3	0.141^b^	**0.001**^**a**^
MSA-C vs. Controls	**< 0.001**^**a**^	**< 0.001**^**a**^
SCA3 vs. Controls	**< 0.001**^**a**^	**0.004**^**b**^
MSA-C within 2 years after onset	22/24 (91.7%)	13/24 (54.2%)
SCA3 within 2 years after onset	2/4 (50.0%)	0/4 (0%)
*P* value	0.086^b^	0.067^b^
MSA-C within 3 years after onset	33/36 (91.7%)	24/36 (66.7%)
SCA3 within 3 years after onset	4/8 (50.0%)	0/8 (0%)
*P* value	**0.014**^**b**^	**0.001**^**b**^

^a^Chi-squared test (post-hoc chi-squared tests adjusted for multiple comparison: *P* < 0.05/3 = 0.0167)

^b^Fisher's exact probability test (post-hoc Fisher's exact probability test adjusted for multiple comparison: *P* < 0.05/3 = 0.0167)

The HCB had a sensitivity of 91.7% for MSA-C and a specificity of 50.0% in patients who underwent MRI within 2 years after disease onset. A grade 2 HCB had a sensitivity of 54.8% and a specificity of 100% for MSA-C. On the other hand, the HCB had a sensitivity of 91.7% for MSA-C and a specificity of 50.0% in patients who underwent MRI within 3 years after disease onset. A grade 2 HCB sign had a sensitivity of 66.7% and a specificity of 100%.

Furthermore, patients with SCA3 had a longer disease duration than patients with MSA-C at the first observation of a grade 2 HCB (11.3 ± 3.5 years vs. 2.3 ± 1.3 years, *p* < 0.001). This was also true when patients with SCA3 were compared with MSA-C at the first observation of either grade of HCB (5.9 years, [1.2–22.5] vs. 1.8 years, [0.2–5.3], *p* < 0.001).

### Orthostatic hypotension

OH 30/15 was observed in 55 of 80 patients with MSA (68.8%), 27 of 41 with MSA-C (65.9%), and 28 of 39 with MSA-P (71.8%). A head-up tilt test within 2 years after disease onset demonstrated OH 30/15 in 26 of 44 patients with MSA (59.1%), 15 of 25 with MSA-C (60.0%), and 11 of 19 with MSA-P (57.9%). Similarly, on testing within 3 years after disease onset, OH 30/15 was observed in 40 of 62 patients with MSA (64.5%), 23 of 35 with MSA-C (65.7%), and 17 of 27 with MSA-P (63.0%).

However, OH 20/10 was reported in 70 of 80 patients with MSA (87.5%), 36 of 41 with MSA-C (87.8%), and 34 of 39 with MSA-P (87.2%). On testing within 2 years after disease onset, OH 20/10 was noted in 35 of 44 patients with MSA (79.5%), 21 of 25 with MSA-C (84.0%), and 14 of 19 with MSA-P (73.7%). On testing within 3 years after disease onset, OH 20/10 was observed in 54 of 62 patients with MSA (87.1%), 32 of 35 with MSA-C (91.4%), and 22 of 27 with MSA-P (81.5%).

## Discussion

Our study demonstrated that HCB (either a vertical or cruciform hyperintensity) appeared both in MSA-C and SCA-3, but not in controls. A grade 2 HCB was found more frequently in MSA-C than in SCA3, whereas there was no difference in frequency of grade 1 or 2 HCB between MSA-C and SCA3. On the other hand, among the patients who underwent MRI within 3 years after disease onset, both grade 2 HCB and grade 1 or 2 HCB were found more frequently in MSA-C than in SCA-3. The HCB had a high sensitivity of 91.7% in the early MSA-C disease course, that is, within 2 years after disease onset. In patients who underwent MRI within 2 or 3 years after disease onset, grade 2 HCB had a high specificity of 100% in differentiating MSA-C from SCA3.

The HCB is a highly sensitive finding in MSA-C, even early in the disease course, as noted in several previous studies. In a longitudinal MRI study of MSA, either a vertical or a cruciform hyperintensity were observed in four patients with MSA-C who had undergone MRI within 3 years after onset [6]. A Japanese cohort study of MSA described cruciform HCB in 64.0% of patients with MSA-C within 2 years and in 87.5% within 4 years after onset of motor impairment, although a vertical hyperintensity (corresponding to grade 1 in our study) was not evaluated [8]. Higashi et al. reported that all 74 patients with MSA-C (disease duration 2.6 ± 1.7 years) had either a pontine midline linear hyperintensity or a complete cruciform HCB [15]. In line with these reports, our study showed that an HCB was frequently observed early in the MSA-C disease course. An HCB of either grade 1 or 2 had a high sensitivity of 91.7% in MSA-C within 2 years after disease onset, and a grade 2 HCB had a sensitivity of 54.2% at 2 years and 66.7% at 3 years after disease onset. Our findings suggest that, in a case of progressive cerebellar ataxia, if an HCB is not observed on MRI within 2 years after the onset of cerebellar symptoms, it is unlikely that the diagnosis is MSA-C. A recent study showed that either a vertical or a cruciform hyperintensity was detected in 80.1% (149/186) of the patients with MSA-C within 3 years after onset [16]. This result is consistent with that of this study but had a lower frequency. This is because the onset symptoms in the previous study included motor symptoms and autonomic nervous symptoms, and HCB is less likely to be observed within 3 years of onset in cases where autonomic disturbances precede cerebellar symptoms. In striking contrast to our study, however, others have reported a low sensitivity of HCB for MSA-C of 18 to 37% [17–19]. However, those studies did not assess the appearance of a vertical pontine T2WI hyperintensity (grade 1 in our study), nor was there a clear definition of the presence or absence of an HCB. It was also not stated in those studies who evaluated the images for the sign.

In differentiating MSA-C from SCA3, a grade 2 HCB early in the course is a very specific finding for MSA-C. Among patients imaged within 2 or 3 years after onset, grade 2 HCB was observed in none of those with SCA3, yielding a high specificity of 100% for distinguishing between MSA-C and SCA3. In line with the results of this study, previous studies that evaluated HCB in MSA-C and SCA including SCA2, SCA3, SCA7, and SCA8, described that grade 2 HCB was not observed in 39 patients with SCA3 within 3 years after onset [16]. In an evaluation of the HCB in adult cerebellar ataxia that included 33 patients with SCA3 (disease duration 6.3 ± 6.0 years), a pontine midline linear hyperintensity was observed 24 (72.7%), but none had the complete cruciform HCB [15]. In another study of the HCB sign in SCA that included 76 patients with SCA3, half (38) had a pontine midline hyperintensity corresponding to grade 1 in our study, whereas a cruciform HCB was observed in only one [9]. The higher frequency of a grade 2 HCB in patients with SCA3 in our study is likely because the disease in many of those patients was of longer duration than in the previous studies we cited. Thus, the mean disease duration at the first observation of grade 2 HCBs in patients with SCA3 was 11.3 ± 3.5 years, which was considerably later than when the sign appeared in the course of MSA-C. Care must therefore be taken in interpreting this finding in our study because the disease duration at MRI was significantly longer in those with SCA3 than in those with MSA-C.

Although a grade 2 HCB has been reported in several types of SCA [9, 10], our study only included patients with SCA3. Therefore, it is unclear whether a grade 2 HCB might appear earlier in the disease course of other SCA types, such that it might be less helpful in differentiating MSA-C from other SCAs. In particular, a grade 2 HCB was reportedly observed more frequently in SCA2 than in SCA3 [9, 15]. Kim et al. reported that grade 2 HCB was observed in 1 of 36 patients with SCA2 within 3 years after onset [16].

OH is probably not a highly sensitive finding in patients with MSA, especially in the early disease course. On testing within 2 years after disease onset, OH 30/15 was observed in only approximately 60% of patients with MSA in this study. Although some patients with MSA present with OH as the initial symptom [18, 20], OH does not always appear early in the disease. A retrospective study showed that 56% of patients had definite MSA-C and 32% of those with possible/probable MSA-C had a systolic blood pressure decrease of at least 30 mmHg at the initial clinical visit (disease duration 3.4 ± 1.5 years in definite and 4.2 ± 2.7 years in possible/probable MSA-C) [21]. A study of parkinsonian syndromes that were confirmed postmortem stated that symptomatic OH was observed in only 3 of 15 patients with MSA within the first year after disease onset [22].

Our study has several limitations. First, we evaluated the HCB only on T2WI. Others have reported that the HCB is better visualized on proton density-weighted imaging or T2*-weighted imaging [23, 24]. However, because in clinical practice, T2WI is a basic sequence frequently obtained during a brain MRI, we believe that findings of our study regarding the HCB on T2WI are easily applicable in routine practice. Second, we did not have pathology confirmation, so the possibility of misdiagnosis in some of the MSA cases cannot be excluded. Third, we used 1.5 Tesla MRI to evaluate the HCB in this study. The visibility of the HCB with 3 Tesla versus 1.5 Tesla has not been compared. Therefore, we do not know if evaluation with 3 Tesla MRI would improve the visibility of the HCB. Although urinary symptoms are important autonomic symptoms for the diagnosis of MSA, as is OH, the frequency of urinary symptoms by disease duration could not be assessed in this study. Finally, in some cases, the middle cerebellar peduncle was not adequately visualized well enough to be evaluated in the axial images, so that the hyperintensity in the middle cerebellar peduncle could not be evaluated in this study. A hyperintensity in the middle cerebellar peduncle on T2WI is reportedly highly specific for differentiating MSA from other neurodegenerative diseases [5, 16, 17, 25, 26]. Combining the HCB with a hyperintensity in the middle cerebellar peduncle may increase the specificity of MSA diagnosis.

## Conclusions

The HCB is a highly sensitive finding in MSA-C, even during the early stages of the disease. A grade 2 HCB at the early stages of the disease is an extremely specific finding for differentiating MSA-C from SCA3. The occurrence of HCB within 3 years of motor symptom onset should be considered as a supportive feature for diagnosing MSA-C in future diagnostic criteria revisions.

## Data Availability

The complete data are available from the corresponding author on reasonable request.

## Abbreviations

MSA: Multiple system atrophy
OH: Orthostatic hypotension
MRI: Magnetic resonance imaging
HCB: Hot cross bun
MSA-C: Cerebellar subtype multiple system atrophy
SCA: Spinocerebellar ataxia
MSA-P: Parkinsonian subtype multiple system atrophy
